# Influenza Vaccines: Successes and Continuing Challenges

**DOI:** 10.1093/infdis/jiab269

**Published:** 2021-09-30

**Authors:** Tanja Becker, Husni Elbahesh, Leslie A Reperant, Guus F Rimmelzwaan, Albert D M E Osterhaus

**Affiliations:** 1 Research Center for Emerging Infections and Zoonoses, University of Veterinary Medicine Hannover, Hannover, Germany; 2 Artemis One Health Research Foundation, Delft, The Netherlands

**Keywords:** influenza vaccine, influenza vaccine development, correlates of protection, next-generation influenza vaccine

## Abstract

Influenza vaccines have been available for over 80 years. They have contributed to significant reductions in influenza morbidity and mortality. However, there have been limitations in their effectiveness, in part due to the continuous antigenic evolution of seasonal influenza viruses, but also due to the predominant use of embryonated chicken eggs for their production. The latter furthermore limits their worldwide production timelines and scale. Therefore today, alternative approaches for their design and production are increasingly pursued, with already licensed quadrivalent seasonal influenza vaccines produced in cell cultures, including based on a baculovirus expression system. Next-generation influenza vaccines aim at inducing broader and longer-lasting immune responses to overcome seasonal influenza virus antigenic drift and to timely address the emergence of a new pandemic influenza virus. Tailored approaches target mechanisms to improve vaccine-induced immune responses in individuals with a weakened immune system, in particular older adults.

Influenza viruses have been recognized as the causative agents of significant respiratory infections in humans for centuries, with recorded pandemics being described as early as the 16th century [1]. Over the last century, yearly epidemics and several pandemics that result in heavy health, societal, and economic burdens have highlighted the significant global impact of influenza viruses. Annual epidemics of seasonal influenza result in the deaths of between 291 000 and 645 000 people [2]. An estimated 3–5 million people suffer from severe respiratory disease caused by seasonal influenza viruses every year. Absenteeism and socioeconomic consequences of the yearly epidemics further cause nonnegligible productivity losses and strain health care capacity.

Influenza viruses are members of the Orthomyxoviridae family. These are enveloped RNA viruses with a segmented genome of 8 single-stranded negative-sense RNA segments. These encode the envelope glycoproteins hemagglutinin (HA) and neuraminidase (NA), the nucleoprotein (NP), the matrix protein (M1) and ion channel protein (M2), the polymerase subunits (PA, PB1, and PB2), the nonstructural protein (NS1), the nuclear export protein (NEP), and the more recently discovered PB1-F2, PB1 N40, PA-X, and M42 proteins [3]. While influenza B viruses are found in humans only, with some indications of spill-over to pigs and seals [4], influenza A viruses have a wide distribution in the animal kingdom, with their natural reservoirs in wild water birds. Avian influenza A viruses are classified into subtypes based on their surface glycoproteins, HA and NA. 16 HA and 9 NA subtypes have been identified in avian species and an additional 2 HA and 2 NA subtypes have been recently discovered in bats. Avian influenza viruses from wild birds may sporadically cross the species barrier to domesticated birds and mammals, like poultry, pigs, horses, and dogs. In these species they may become established pathogens. In poultry, such adapted viruses are classified by their pathogenicity in chickens into high- and low-pathogenicity avian influenza viruses (HPAIV and LPAIV, respectively; for review see [5]).

Zoonotic infections from domestic pigs and poultry have caused numerous human influenza cases, which usually are not or only poorly transmissible between humans. Human infections with H5 HPAIV, H7 LPAIV, H7 HPAIV, and H9 LPAIV of poultry have caused many cases of influenza with high fatality rates in recent years. The further adaptation of these viruses to replication in, and transmission among, humans may lead to the eventual development of a pandemic virus. Four influenza pandemics have emerged in the past century, causing the Spanish flu (1918, H1N1), the Asian flu (1957, H2N2), the Hong Kong flu (1968, H3N2), and the swine-origin flu pandemic of 2009 (H1N1/09). Although these viruses eventually have all originated from wild avian reservoirs, new genetically reassorted viruses typically emerged in domestic pigs and poultry, before initiating pandemics in humans.

Upon their introduction, pandemic viruses can spread rapidly and circulate among a virtually naive and susceptible human population that has not been previously exposed to an antigenically similar influenza A virus, often resulting in devastating morbidity and mortality. Collectively, the 4 pandemics of the last century have resulted in more than 50 million human fatalities. These pandemic viruses all have continued to circulate after their respective pandemics were over and typically replaced one of the previously circulating seasonal influenza A viruses. The new seasonal influenza viruses gradually drift genetically and antigenically, escaping from antibody-mediated virus neutralizing immunity that builds up in the population upon their annual reappearance. The virus neutralizing immunity that drives seasonal influenza genetic and antigenic drift appears predominantly directed against the globular head of the virus HA protein. As a result, this glycoprotein continuously accumulates mutations that eventually affect recognition by the existing neutralizing antibody landscape across the population. The continuous antigenic drift of seasonal influenza viruses requires regular updates of seasonal influenza vaccines in order to correctly match the circulating viruses [6].

In the interpandemic periods, annual seasonal influenza epidemics, which typically occur in and around winter months in temperate climate zones, collectively have resulted in at least an equivalent number of fatal cases as the 4 past pandemics combined. Severe seasonal influenza infections and complications mainly occur in the so-called high-risk groups, such as older adults, people with chronic disease or impaired immunity, pregnant women, and young children. Therefore, these groups are the first targets of annual influenza vaccination programs, as advised by the World Health Organization (WHO) as well as national and regional public health agencies, such as the US Center for Disease Control and Prevention (CDC), although their response to vaccination is often suboptimal. Because of their contacts with high-risk groups, health care workers also are advised to be annually vaccinated against seasonal influenza.

An estimated 2%–10% of vaccinated, healthy individuals do not produce adequate levels of antibodies following vaccination [7]. This may be due to their genetic characteristics (eg, human leukocyte antigen [HLA] type or single-nucleotide polymorphisms) [8] or to the state of their immune system. In apparently healthy vaccinees, the immune response to vaccination can be influenced negatively by lifestyle (eg, stress, nutritional deficiency, or obesity [9–15]), previous contact with closely related viruses [16] or age-related changes of the immune system (immunosenescence [17–20]). Changes in the immune system induced by comorbidities (eg, diabetes), immunosuppression, or medication can further weaken the immune response to vaccination. High-risk group populations are increasing as society ages, diseases of affluence rise, and people with chronic diseases live longer due to better health care, particularly in developed countries. These individuals not only have an increased risk of vaccination failure but also face worse disease outcomes in the event of vaccination failure. Vaccine improvements for these risk groups are therefore direly needed.

Nevertheless, vaccination is the most cost-effective way to prevent influenza virus infections. Until recently, all influenza vaccines were generated in embryonated chicken eggs, based on a technology that was developed in the middle of the 20th century. Although this proved to be a quite efficient method to produce high virus concentrations, it has shown major shortcomings calling for new generation influenza vaccines. Currently available seasonal influenza vaccines are egg- and cell-based inactivated influenza vaccines (IIVs), a live attenuated influenza vaccine (LAIV), and a baculovirus recombinant HA vaccine that is produced in insect cells (Table 1). The development of universal influenza vaccines that would provide both a broad and a long-lasting protection against preferably all circulating and emerging influenza A and B subtypes and variants, including pandemic viruses, with robust responses also induced in high-risk groups, is one of the greatest challenges of modern vaccinology. These will undeniably benefit from current and emerging knowledge of correlates of protection against influenza and of the broad spectrum of novel technologies and technology platforms that are currently used and explored in modern vaccine development, including as part of ongoing responses against the COVID-19 pandemic.

**Table 1.** Overview of Currently Used Vaccine Types on the US and European Market

Vaccine type		Produced in	Adjuvants	Administration route	HA per strain [µg/dose]
**Inactivated**	Subunit	Egg	None	Intramuscular/subcutaneous	Usually 15 µg/strain/dose
	Subunit	Egg	MF59^a^	Intramuscular	Usually 15 µg/strain/dose
	Subunit	Cell culture	None	Intramuscular	Usually 15 µg/strain/dose
	Split	Egg	None	Intramuscular/subcutaneous	Usually 15 µg/strain/dose
	Split/high-dose^a^	Egg	None	Intramuscular	60 µg/strain/dose
**Live attenuated**		Egg	None	Intranasal	10^6.5–7.5^ FFU/strain/dose
**Recombinant**		Cell culture	None	Intramuscular	45 µg/strain/dose

Adapted and modified from [21, 22].

Abbreviations: FFU, fluorescent focus units; HA, hemagglutinin.

^a^Recommended for people aged ≥ 65 years.

## INFLUENZA VACCINE DEVELOPMENT—A GLOBAL ACHIEVEMENT

**Figure 1.**
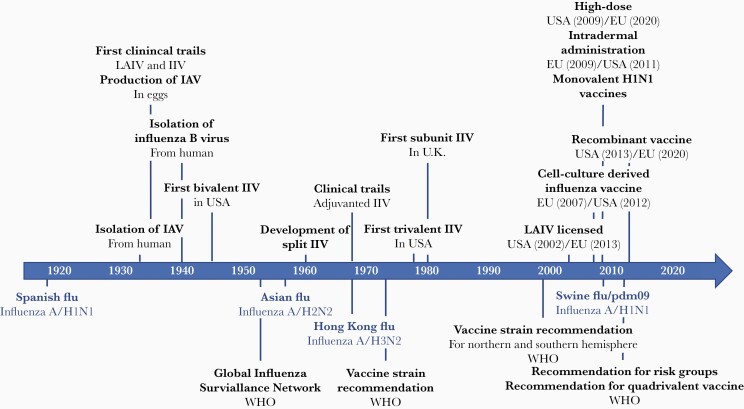
Timeline of influenza vaccine history [23–29]. Abbreviations: IAV, influenza A virus; IIV, inactivated influenza vaccine; LAIV, live attenuated influenza vaccine; WHO, World Health Organization.

The history of influenza vaccination is a success story that started almost a century ago (Figure 1). The first influenza vaccines were a monovalent inactivated influenza A vaccine produced in embryonated chicken eggs and a live-attenuated vaccine in the mid-1930s [30, 31], only a few years after the first isolations of influenza viruses from pigs and humans, respectively [32, 33]. Influenza B virus was discovered in 1940. The first bivalent vaccine containing 1 influenza A and 1 influenza B strain and was produced and tested by the US army from 1942 onward. It became available for the general population in the United States by 1945. Split and subunit vaccines were subsequently developed beginning in the 1960s. Trivalent vaccines, incorporating 2 influenza A subtypes and 1 influenza B strain, became available in 1978. It was not until 2012 that the first quadrivalent vaccines incorporating 2 influenza A subtypes and 2 influenza B strains was approved by the US Food and Drug Administration (FDA). In the following year, the first recombinant HA vaccine expressed by insect cells was licensed in the United States (for a detailed review of the history of influenza vaccination see [23, 24]). The 2009 H1N1 influenza pandemic was the first pandemic for which a specific pandemic influenza vaccine became globally available. Although it came late for the southern hemisphere, the pandemic vaccine was shown to effectively prevent laboratory-confirmed influenza, related hospitalization, and mortality in the northern hemisphere [34]. Likewise, annual vaccination of the general population and especially of high-risk groups with seasonal vaccines, before the start of the influenza season, has been shown to considerably reduce morbidity, mortality, and economic losses associated with influenza [35]. Economic losses averted by influenza vaccination are related to reduced health care costs and maintained productivity (eg, [36]). As a recent example, the CDC estimated for the United States the prevention of an estimated 7.52 million illnesses, 3.69 million medical visits, 105 000 hospitalizations, and 6300 deaths due to influenza, by influenza vaccination during the 2019–2020 season [37]. Besides direct protective effects of influenza vaccination in the vaccinees, indirect effects in other members of the community may be observed due to reduced virus circulation. For example, a governmental vaccination program for schoolchildren in Japan from 1962 to 1994 was linked to a drop of excess mortality associated with influenza and pneumonia in the elderly, which was not observed after the program was discontinued. The vaccination coverage in adults was reported lower than in children, indicating an indirect protective effect of the vaccination of children on the health of the elderly [38]. The effectiveness of influenza vaccination in older adults is lower than that in younger adults [39, 40]. The efficacy and effectiveness in the elderly was even initially questioned by a Cochrane meta-analysis ([41]; updated version available [42]). However, methodologically adjusted meta-analysis resulted in values ranging from 30% to 50% vaccine effectiveness, largely depending on the age of the vaccinees and the matching of the vaccine strains with the circulating influenza viruses [43]. Furthermore, less severe disease is typically reported in vaccinated patients than in nonvaccinated patients hospitalized with laboratory-confirmed influenza [44]. Interestingly, influenza vaccination may reduce the impact of other respiratory infections, which can occur as coinfections during influenza, as well as counter the rise of antimicrobial resistance [45]. Maintaining or increasing influenza vaccination coverage during the currently ongoing COVID-19 pandemic has been recommended by public health agencies to prevent additional seasonal influenza burden on the highly strained health care systems. However, both excess mortality data and viral surveillance have revealed limited cases of other respiratory infections, and in particular influenza, during the COVID-19 pandemic compared to previous seasons [46, 47]. The limited circulation of seasonal influenza viruses may be mainly due to the set of nonpharmaceutical public health interventions deployed in most countries since the beginning of the pandemic (eg, increased hygiene measures, face masks, social distancing, and contact reduction). While the effectiveness of such measures against both severe acute respiratory syndrome coronavirus 2 (SARS-CoV-2) and influenza virus infections is undeniable, their implementation is temporary due to considerable social and economic consequences. Effective vaccination remains the preventive measure of choice against influenza.

## CORRELATES OF PROTECTION

**Figure 2.**
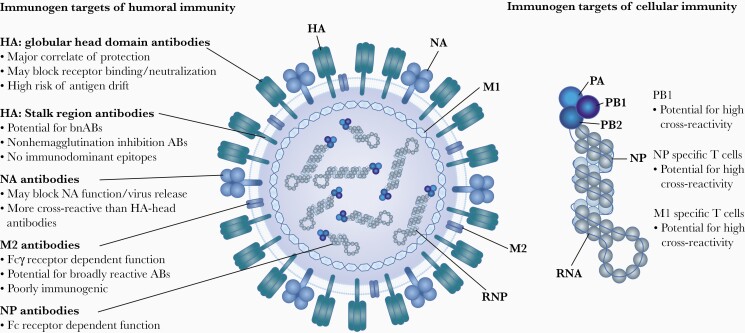
Major immunogens of influenza virus. Abbreviations: AB, antibody; bnAB, broadly neutralizing antibody; HA, hemagglutinin; M, matrix protein; NA, neuraminidase; NP, nucleoprotein; P, polymerase; RNP, ribonucleoprotein. Adapted from [49, 50] and created with BioRender.com.

Remarkable advances in our understanding of the correlates of immune protection against influenza increasingly point towards the possible development of improved and more broadly protective vaccines against seasonal and pandemic influenza. In addition to animal and cell culture-based models of influenza, human challenge studies have made significant contributions to the identification of correlates of protection (see eg, [48]). A number of these high-profile induced immunity targets (Figure 2) are actively pursued in current cutting-edge influenza vaccine research and development efforts around the world.

### Antibodies

#### Neutralizing HA-Specific Antibodies

It is generally accepted that antibodies directed against the globular head domain of the influenza virus HA are a major correlate of protection. The HA is the viral receptor binding protein allowing attachment of virus particles to cellular targets, before endocytosis, fusion of viral and cellular membranes, and actual infection occur. Antibodies directed to epitopes located in or in close proximity to the receptor-binding site (RBD) can prevent binding of the virus to its receptor, thereby neutralizing the virus before cellular infection takes place. When serum titers of virus strain-specific HA antibodies, induced by vaccination or infection, are high enough, they protect subjects from subsequent (re)infection. The protective effect of virus-neutralizing HA-specific antibodies has been demonstrated both in experimentally infected animals and humans [51]. The induction of HA-specific antibodies is used as a surrogate of vaccine efficacy. Seasonal influenza vaccines are registered every year provided they fulfill the minimal requirements of national or regional medicine agencies (like the FDA or the European Medicines Agency) on the serological outcome of vaccination and potency of the vaccine (with >15 µg HA per vaccine strain). Most commonly, the serological outcome of vaccination is measured with the hemagglutination inhibition (HI) assay, which is a validated proxy for virus neutralization.

For optimal vaccine efficacy, it is essential that the vaccine strains antigenically match the epidemic strains closely. Because the HA of seasonal influenza virus strains undergo continuous antigenic drift, seasonal influenza viruses eventually evolve to escape from recognition by virus-neutralizing antibodies, necessitating the update of the vaccine strains almost annually [6, 52].

#### NA-Specific Antibodies

The NA is the other major viral envelope protein and functions as a receptor-destroying enzyme, which is crucial for efficient release of virus from infected cells during the last stages of the virus replication cycle. Antibodies directed against the enzymatic site of NA can block its function and can contribute to protective immunity. This has been demonstrated in various in vitro and in vivo systems [53–62]. In contrast to HA-specific antibodies, NA-specific antibodies cannot prevent infection, but limit the release and further spread of virus from infected cells, and thus improve the clinical outcome of the infection. It is of interest to note that NA-specific antibodies tend to be more cross-reactive than antibodies specific for the HA head domain [63, 64], although NA also displays antigenic drift [65, 66]. The enzyme-linked lectin assay has emerged as a suitable assay for the detection of NA-specific serum antibodies and the antigenic characterization of NA [65, 67].

#### Other Non-HI Broadly Reactive Antibodies

Other antibodies that may contribute to broadly protective immunity include those directed against conserved proteins like M2 [68], NP, NA [69–71], and the stalk region of HA [72, 73], which have therefore been considered as potential (universal) influenza vaccine antigens.

The M2 protein is a minor antigen on virus particles but is abundantly expressed on virus-infected cells. Compared to HA and NA, M2 is poorly immunogenic because antisera raised against the virus typically contain few M2-specific antibodies. The protective effect of M2-specific antibodies has been demonstrated after hyperimmunization and passive administration of these antibodies in animal models [68] and was shown to be dependent on Fcγ receptors [74]. This indicates that antibody-dependent cellular cytotoxicity (ADCC) by natural killer (NK) cells or neutrophils, or antibody-dependent phagocytosis by macrophages likely play a role in conferred protection [74, 75].

A protective effect of NP-specific antibodies has been demonstrated in mice [76, 77], although the underlying mechanism remains unclear. The effect was dependent on Fc receptors and CD8^+^ T cells. Therefore, it has been suggested that formation of NP immune complexes and opsonization play a role in protection [76, 77], although this could not be confirmed in vitro [78].

The identification of virus-neutralizing antibodies directed to the stalk region of the trimeric HA molecule, has attracted a lot of attention [72], because compared to the variable head domain, the stalk region is relatively conserved, opening avenues for strong and broader immune responses. In contrast to virus-neutralizing antibodies specific for the head domain, which defines HA’s antigenic properties and contains the RBD, stalk-specific antibodies fail to inhibit agglutination of erythrocytes and are therefore referred to as non-HI antibodies. Alternative mechanisms independent of blocking receptor binding account for their protective effect. These include preventing HA conformational changes in the endosomes and subsequent fusion of the virus membrane with the endosomal membrane, thus preventing release of the viral genome into the cytosol [79], effects on virus egress from infected cells [80], and interference with HA maturation by preventing its cleavage by host proteases [72]. Interactions between the Fc region of broadly neutralizing HA stalk-specific antibodies and Fcγ receptors were found to be essential in protecting mice from lethal influenza virus challenge. This suggests that ADCC by HA stalk-specific antibodies contributed to protection [81]. The development of standardized assays for the detection and quantification of unconventional non-HI antibodies is essential and the subject of an active area of research, in order to determine minimal antibody titers required for protection and compare potency between studies.

#### Mucosal Antibodies IgA

The production of polymeric immunoglobulin A (pIgA) and the subsequent transcytosis across the epithelium after binding to the polymeric Ig receptor (pIgR) yields secretory IgA (sIgA), a complex consisting of dimeric IgA and the secretory component, which is a cleavage product of the pIgR [82].

sIgA is more efficient than IgG or monomeric IgA for inhibiting influenza virus entry [83]. Most vaccines are subunit, split virion, or whole inactivated preparations that are administered intramuscularly. These vaccines induce good serum antibody responses but limited local mucosal antibody responses. In contrast, the use of live attenuated vaccines, which are administered topically, induces efficient virus-specific IgA responses, but possibly more limited serum antibody responses. While the clinical effectiveness of both types of vaccines is similar, the immune correlates of protection differ [84].

### Virus-Specific T Cells

It has been demonstrated in various animal models, including mice [85–88] and nonhuman primates [89], that virus-specific T lymphocytes, in particular CD8^+^ T cells, are an important correlate of protection against influenza virus infections and contribute to heterosubtypic immunity (reviewed in [88]). Because the majority of virus-specific CD8^+^ cytotoxic T lymphocytes (CTL) recognize conserved internal proteins, like NP and M1 [90, 91], they are highly cross-reactive [92–95]. Indeed, CTL induced after infection with seasonal H1N1 and H3N2 influenza virus cross-react with influenza A viruses of the H5N1 subtype [96, 97], H7N9 subtype [93], H1N1pdm09 viruses [94, 98], and swine origin variant H3N2 viruses [94].

A protective role for cross-reactive virus-specific CTL in humans was first shown after experimental infections [99]. In the absence of virus-specific antibodies to the challenge virus, the lytic activity of peripheral blood mononuclear cells inversely correlated with the extent of virus shedding. More recently, it was demonstrated that the frequency of preexisting cross-reactive CD8^+^ T cells inversely correlated with disease severity in patients infected with the pandemic H1N1 virus of 2009 [100, 101]. In acutely infected patients, it was demonstrated that the anamnestic cross-reactive virus-specific CD8^+^ CTL response was very rapid, which may have contributed to accelerated clearance of the virus [95]. Furthermore, in patients infected with the avian H7N9 virus, positive disease outcome correlated with the magnitude of the virus-specific CD8^+^ T-cell response [102]. The observation that CTL epitopes accumulate amino acid substitutions at anchor or T-cell receptor residues that are associated with escape from recognition by CD8^+^ T cells further support the notion that CTL control influenza virus replication and exert selective pressure on the virus [103]. In a human challenge study, virus-specific CD4^+^ T lymphocytes were also shown to correlate with reduced disease severity [104].

## CURRENTLY AVAILABLE INFLUENZA VACCINES

The currently available seasonal influenza vaccines (Table 1) provide protection against circulating virus strains that are closely related to those represented in the vaccine but fail to provide long-lasting and broadly protective immunity against more distantly related drifted influenza viruses. This has led to the development of a procedure of influenza vaccine strain selection that is coordinated by the WHO twice a year in close consultation with an international network of key laboratories and academies to review surveillance, clinical study results, and the availability of vaccine viruses [105]. For a few decades, this strain selection was used to produce trivalent vaccines that represented the 2 circulating influenza A virus subtypes and 1 influenza B virus lineage. Since 2013–2014, mainly quadrivalent influenza vaccines are administered. They represent 2 circulating influenza A virus subtypes and 2 influenza B virus lineages: the Yamagata and Victoria lineages, which display limited serum cross-reactivity. As cross-B–lineage protection appears to be related to the level of exposure to influenza B virus, which increases with age, protection against the seasonal influenza B virus lineage absent from trivalent vaccines may occasion vaccine failure in children. Quadrivalent vaccines were shown to provide improved protection against influenza B virus in children, which are less likely to have preseasonal immunity in case of a B linage mismatch of a trivalent vaccine [106].

Nevertheless, these seasonal vaccines provide little or no protection against zoonotic or pandemic influenza viruses. Thus, upon the emergence of a pandemic, an update of the vaccine with the pandemic virus strain is necessary before the deployment of vaccination programs can occur. This requires swift vaccine development, which is hampered by the current production approach used for the vast majority of seasonal influenza vaccines and based on the use of embryonated chicken eggs. While this technology is relatively efficient and cost-effective, it is associated with a number of major disadvantages. First, generating vaccine candidate viruses by reassortment, and to a lesser extent by reverse genetics, that are highly productive in eggs, is highly time-consuming. The development time of seed viruses, using the backbone of an egg-adapted virus and expressing the HA and NA genes of the circulating viruses, and the subsequent vaccine production time may take as long as 6 to 8 months before the first vaccine doses become available. During this time lapse, new drift variants of seasonal and pandemic influenza viruses alike may arise, resulting in vaccine mismatch. Adaptation of the vaccine seed viruses to replication in avian tissue may also lead to adaptive mutations that may result in yet further mismatch with circulating strains. Such changes may severely reduce vaccine-induced protection [107]. Finally, the capacity of the egg production system requires careful planning, as it cannot be scaled-up within a short period of time for obvious reasons, as it depends on laying chickens. The system is furthermore vulnerable to the risk of avian influenza and other poultry disease outbreaks that may paralyze the supply of embryonated chicken eggs.

Although the relatively cost-effective egg-based vaccine production platform allows the production of more than a billion vaccine doses annually, several vaccine manufacturers are addressing these shortcomings by the use of accredited cell lines, like African green monkey kidney (Vero) cells, Madin-Darby canine kidney (MDCK) cells, and others as new platforms to produce vaccine viruses at yields that are comparable to those obtained in eggs [108, 109]. Several of the disadvantages of the egg-based production platform, like lack of scalability, avian mutation-based mismatch, and vulnerability to avian disease outbreaks may at least in part be overcome by using these new production platforms. The price of cell culture-produced IIVs, however, remains considerably higher than that of their egg-based counterparts.

### Inactivated Influenza Vaccines

Among the seasonal influenza vaccines that are most frequently used today, are the IIVs produced in embryonated chicken eggs. These vaccines have an excellent safety record. Essentially 3 types of IIVs are being used today, based on whole-virions, split-virions, and HA and NA subunits [110].

Classically, alum and oil-in-water emulsions (eg, MF59) have been used as adjuvants in some of the nonreplicating seasonal and pandemic human IIVs. These vaccines can profit from the combined use of adjuvants by the resulting increase of the specific immune response upon vaccination, or alternatively by reducing the antigen content in a dose sparing way. The latter may particularly be of interest during an influenza pandemic, when production capacity of vaccine antigen may become a limiting factor for effective vaccination coverage.

In addition to adjuvants, high-dose IIVs are produced to increase vaccine immunogenicity. In contrast to the standard dose IIVs (15 µg HA/strain), high-dose IIVs contain 4-fold HA dose. Trials with individuals aged 65 years and older demonstrated a higher antibody response, a better protection against laboratory-confirmed influenza illness, and a reduced hospitalization rate of nursing home residents when high-dose IIVs instead of the standard vaccine were used [111–113].

### Live Attenuated Influenza Vaccines

LAIVs are based on the use of a cold-adapted virus that replicates well in embryonated chicken eggs and better at temperatures lower than the normal human body temperature. These are largely limited to replication in the upper respiratory tract and are therefore attenuated. LAIVs are generated by reassortment and are composed of the internal genes of a cold- and egg-adapted virus combined with the HA and NA of the respective seasonal influenza viruses identified by the influenza vaccine strain selection. As the immunization is based on replicating vaccine virus administered intranasally, it induces a strong local mucosal IgA response. Importantly, it induces both CD4^+^ and CD8^+^ T-cell responses. To what extent the internal proteins of the cold- and egg-adapted vaccine virus at the origin of CD8^+^ T-cell responses have been subject to escape mutations over time in the circulating viruses is not clear at present [114, 115].

The overall level of protection induced by LAIVs in adults is comparable to that induced by IIVs. However, LAIVs appear to be less effective than IIVs in older adults, while they appear more effective in children. Therefore, the recommended age for this type of vaccine is from 24 months to 49 years of age. The exclusion of children under 2 years of age is related to an increased risk of induction of wheezing. Similarly, because LAIV vaccination depends on replication of the attenuated vaccine virus in the upper respiratory tract, which may result in some mild replication-associated symptoms, certain high-risk groups for influenza and pregnant women also have been excluded.

### Recombinant HA Vaccine

The first purified recombinant HA vaccine FLUBLOK, developed by Protein Sciences and now marketed by Sanofi Pasteur, is formulated into trimer “rosettes,” that are produced in insect cells by a baculovirus expression system. It was shown to be 30% more efficacious than traditional IIVs for adults older than 50 years [116]. This may at least in part be related to a 3-fold higher HA load than classical IIVs. Until now the price of this first recombinant HA vaccine is relatively high, probably due to the limited scale at which it was originally produced.

## APPROACHES TO IMPROVE INFLUENZA VACCINES

### Next-Generation Influenza Vaccines

Next-generation influenza vaccines are urgently needed in order to address seasonal influenza antigenic drift and contribute to better pandemic preparedness [117, 118]. These aim at inducing broader intra- and intersubtypic as well as longer-lasting protective immune responses. Their production aim at rapid and large-scale capacity, overcoming one of the major shortcomings of the embryonated chickens egg system. Major challenges faced by the research and development community for the successful development of next-generation influenza vaccine candidates therefore include the induction of the desired humoral and T-cell–mediated immune responses against conserved epitopes, as well as the development of large-scale production systems. Mammalian cell lines and baculovirus expression system based on insect cells already offer relevant alternatives to the embryonated egg system, although costs will need to align with the latter for competitiveness [119].

Significant progress towards these next-generation vaccine candidates has been achieved worldwide [48, 120, 121]. Vaccine candidates in the development pipeline can be divided into 2 categories (for an overview of influenza vaccines in clinical trials see [122]). The first are designed to elicit broadly neutralizing antibody (bnAb) responses toward highly conserved conformational epitopes in the HA stem [123] and non-virus–neutralizing (non-VN) antibody responses to structurally conserved regions of influenza virus surface membrane proteins (HA, NA, and matrix protein 2 ectodomain [M2e]). The second are designed to induce cross-protective T-cell responses against predominantly internal proteins, like M1, NP, and PB1 [124]. The respective immune responses contribute largely to preventing infection on the one hand, and reducing disease severity upon infection on the other.

The recent discovery of bnAbs against influenza viruses indicates that the generation of a broadly protective vaccine may indeed be attainable. The majority of these bnAbs, however, are directed toward highly conserved conformational epitopes in the HA stem, which lack the immunodominance of epitopes displayed by the influenza HA head. A key strategy proposed to avoid or circumvent influenza HA head immunodominance is by generating recombinant headless HA proteins (or HA stems). However, removal of the transmembrane domain on the one hand and HA head on the other without extensive compensatory modifications to stabilize the remaining molecule leads to loss of native conformation of the HA stem, resulting in low if any presence of conformational bnAb-inducing epitopes. This has led to several approaches currently pursued towards stabilization of HA stems [125].

The HA head is immunodominant, with immune responses naturally targeting the antigenically variable region that surrounds the RBD. However, head-specific bnAbs have also been shown to be induced upon infection. Harnessing HA head immunodominance and steering head-specific immune responses toward more conserved regions of the HA head represents a promising complementary approach. Among explored vaccination strategies to induce reactive antibodies against conserved HA epitopes is the use of sequential vaccination with different chimeric HAs displaying the same HA stem and different HA heads [125].

In addition to HA, several studies have shown that inclusion of NA into influenza vaccines enhances the protective efficacy of these vaccines [126]. Serum antibodies that can inhibit NA activity are known to correlate with protection against human influenza independently of HA-specific antibodies [127, 128]. M2e also is a safe and broadly protective influenza A vaccine antigen that primarily protects by antibody-dependent effector mechanisms. M2e is naturally a tetramer and thus can present quaternary epitopes to which antibodies with very high affinity may bind. Immune responses directed against M2e are nonetheless very weak following natural infection and virtually absent following vaccination with any of the licensed influenza vaccines [129]. Several strategies to overcome the inherent problems of M2e limited immunodominance are currently being explored [130].

The role of MHC class I restricted CD8^+^ T cells in accelerating virus clearance and limiting disease severity upon reinfection with influenza virus is increasingly recognized to potentially play a significant contribution to vaccine efficacy and breadth of protection. The induction of cellular-mediated immunity is largely dependent on replicating viruses and thus, in addition to LAIVs, viral vectored vaccines, as well as DNA- and RNA-based vaccines and virus-like particles are promising new technology platforms towards broader influenza vaccines [131]. In particular, the use of viral vectors for the presentation and delivery of (modified) vaccine antigens offer many advantages, in terms of both safety and efficacy (for review see [132, 133]). As an example, modified vaccinia Ankara (MVA) is a highly attenuated and replication-deficient strain of vaccinia virus that is increasingly used in biomedicine for vaccine development. The induction of protective humoral and cellular immune responses by MVA against a wide range of viruses [133], including influenza viruses in animal models [134–136] and in humans (phase 1/2a clinical trial [137, 138]), has been widely demonstrated. The safety and immunogenicity of MVA expressing influenza virus proteins was furthermore confirmed in elderly persons [139]. MVA vector vaccines rapidly induce strong antigen-specific CD4^+^ T helper cell as well as CD8^+^ effector T-cell immunity, leading to robust and durable protective immune responses. The presentation of viral targets of both humoral and cell-mediated immunity by MVA and other viral vectors has strong potential to optimize and synergize the induction of broad immune responses against influenza.

The striking success of mRNA vaccines against COVID-19 including in high-aged individuals [140] highlights the promise this technology might hold for the development of future influenza vaccines. Indeed, several approaches based on nonreplicating or self-replicating mRNA encoding for influenza HA, NP, and/or M1 have been developed and have demonstrated the general capacity of influenza mRNA vaccines to induce humoral and cellular immune responses and to provide protection against homologous and heterologous strains in animal models [141–143]. So far, only lipid nanoparticles with nucleoside modified mRNA encoding the full-length, membrane-bound form of HA from H10N8 (A/Jiangxi-Donghu/346/2013; NCT03076385) or H7N9 (A/Anhui/1/2013; NCT03345043) by Moderna Therapeutics have reached clinical trials. While they were well tolerated and induced humoral immune responses, no cell-mediated responses were detected [144]. Due to the progress and success in the development of mRNA vaccines against SARS-CoV2 as well as several advantages compared to standard egg-based technologies, a further focus on this area can be expected.

### New-Generation Adjuvants

Adjuvants can improve the vaccine response by enhancing and modulating the immune response. In general, adjuvants act through different mechanisms or a combination thereof. They can create an antigen depot, activate the innate immune response, induce inflammasomes and cytokines, recruit immune cells, improve antigen uptake, enhance immune cell maturation, and change the activation profile of adaptive immune cells (reviewed in [145]). Up to now, only 6 adjuvants have been licensed in combination with influenza vaccines (Alum, MF59, AS03, AF03, virosomes and heat labile enterotoxin) but not all are currently in use [25]. Adjuvanted influenza vaccines were reported to generally improve humoral and cellular responses as well as the immune response in risk groups like the elderly and children [146–149].

For instance, multiple studies indicate that the addition of the oil-in-water adjuvant MF59 leads to a faster and higher antibody induction and a better cellular immune repose compared to nonadjuvanted influenza vaccines [146, 148, 150, 151]. MF59 was reported to provoke proinflammatory cytokines and chemokines as well as chemoattractants (eg, CCL2, CCL3, and CXCL8) and to contribute to the recruitment, activation, and maturation of antigen-presenting cells (eg, dendritic cell and macrophages) at the injection site [25, 152]. Several human clinical trials have investigated influenza vaccines with experimental adjuvants (reviewed in [25]). The addition of a suitable adjuvant typically aims at altering the immune response in favor of an immune response type that correlates with protection. For instance, Toll-like receptor (TLR) ligands and agonists (eg, imiquimod [TLR7], resiquimod [TLR7/8], or CpG oligodeoxynucleotids/immunostimulatory sequences [TLR9]) were associated with CTL activation [25, 122, 153, 154], which might be necessary to improve vaccination outcome in risk groups. Indeed, a topical pretreatment with the TLR7 agonist imiquimod before an intradermal influenza vaccination was shown to significantly increase the immunogenicity of the vaccine in the elderly with chronic diseases [155] and to induce protection against a heterologous influenza strain in a phase 2b/3 trial [156].

### Immunomodulators

Kinases are one class of biological response modifiers that has been investigated for intervention strategies against influenza viruses. Host kinases not only regulate influenza virus entry and replication, but are also integral components of various antiviral and inflammatory pathways allowing them to shape the immune response (reviewed in [157] and [158]). The therapeutic potential of targeting kinases has long been recognized in the field of oncology. While small-molecule kinase inhibitors (SMKIs) have been primarily used in cancer therapy, some are also applied in nonneoplastic diseases such as chronic inflammatory diseases (eg, rheumatic arthritis, Crohn disease, or ulcerative colitis) [159, 160]. A large number of SMKIs are FDA approved or in development [161], many of which have been shown to regulate the immune response. Although immunomodulatory effects of SMKIs have been mainly described in the context of cancer models or patient trials, there is a strong potential that they can also improve immune responses upon vaccination, especially in risk groups. Several studies have described the inhibition and/or inactivation of suppressive immune cells like regulatory T cells (T_regs_) and myeloid-derived suppressor cells by different kinase inhibitors [162–165]. Together with the inhibition of T_regs_, an increase in the effector T cells has the potential to lead to an improved ratio favoring immune stimulation [162, 166]. Some were shown to contribute to the optimal priming of CTLs and NK function by favoring T helper 1 cells.

SMKIs have been successfully used as influenza vaccine adjuvants in several murine vaccination/challenge experiments. Topical application of epidermal growth factor receptor inhibitors (EGFRIs) before intradermal vaccination increased the humoral and cellular vaccination response and led to a reduced viral load in the lungs and improved survival rates in challenged mice [147]. Systemic EGFRI treatment of cancer patients was correlated with increased cytokine expression, immune cell recruitment, and T_reg_ inhibition in the skin, suggesting a change of the immune homeostasis in favor of enhanced immune reactions to vaccines [147]. Another study, by Lanna et al, investigated the capacity of mitogen-activated protein kinase inhibitors to overcome immune senescence and improve responses to influenza vaccination in aged mice [167]. A reversion of immunosenescence by SMKIs was observed and characterized by an upregulation of CD27 and CD28 as well as telomerase reexpression, increased T-cell proliferation, interleukin-2 production, and cytotoxicity, and improved T- and B-cell functions [167]. These results indicate that SMKIs not only may restore the impaired functions of a senescent immune system, but they can also reverse other immune dysregulations attributed to inflammaging or stress [14].

## Conclusions

Although influenza vaccination is a global achievement and has considerably contributed to reducing morbidity and mortality associated with influenza worldwide, there is an urgent need for novel technologies and strategies to improve influenza vaccine responses towards broader and longer-lasting protective immunity, including in individuals at risk of vaccine failure. The proportion of the population with expected poor vaccine responses is increasing, especially in developed countries. Although there are already strategies to improve vaccine responses for some risk groups, for example the elderly [168, 169], additional knowledge of the mechanisms that lead to vaccine failure in risk groups and ways to overcome them through immune modulation may further contribute to improving vaccine design. Such rational approaches to next-generation influenza vaccine development will ultimately help to design new or improved vaccines tailored to induce the range of correlates of protection ensuring broader cross-reactive protection, as well as to address the needs of those most vulnerable to severe disease outcomes.

## Notes

***Financial support*.** This work was supported by the Alexander von Humboldt Foundation in the framework of the Alexander von Humboldt Professorship endowed by the German Federal Ministry of Education and Research; and by the European Union’s Horizon 2020 Research and Innovation Program (grant numbers 848166 [ISOLDA] and 874650 [ENDFLU]).

***Supplement sponsorship.*** This supplement is sponsored by the Bill and Melinda Gates Foundation.

***Potential conflicts of interest.*** All authors: No reported conflicts of interest. All authors have submitted the ICMJE Form for Disclosure of Potential Conflicts of Interest. Conflicts that the editors consider relevant to the content of the manuscript have been disclosed.
